# The association between stressful life events and depressive symptoms among Cypriot university students: a cross-sectional descriptive correlational study

**DOI:** 10.1186/1471-2458-13-1121

**Published:** 2013-12-05

**Authors:** Sokratis Sokratous, Anastasios Merkouris, Nicos Middleton, Maria Karanikola

**Affiliations:** 1 Department of Nursing, Faculty of Health Sciences, Cyprus University of Technology, Vragadinou Street, Limassol, Cyprus

**Keywords:** Center for epidemiology studies (CES-D), Depression, Life events scale for students (LESS), Stressful life events, Cypriot university students

## Abstract

**Background:**

Previous findings suggest that stressful life events have a causal relationship with depressive symptoms. However, to date little is known concerning the contribution of the number and severity of recent stressful life events on the prevalence of depressive symptoms among university students. The aim of this study was to investigate the prevalence of depressive symptoms and its association with the number and the severity of self-reported stressful life events among university students in Cyprus.

**Methods:**

A descriptive correlational design with cross sectional comparison was used. The CES-D scale was applied for the assessment of depressive symptoms and the LESS instrument for stressful life events. Both scales were completed anonymously and voluntarily by 1.500 students (response rate 85%).

**Results:**

The prevalence of mild to moderate depressive symptoms [CES-D score between 16 and 21] and of clinically significant depressive symptoms [CES-D score ≥ 22] were 18.8% and 25.3% respectively. There were statistically significant differences in clinically significant depressive symptoms by gender, with higher rates among women (x^2^ = 8.53, df = 1, p = 0.003). Higher scores on the LESS scale were associated with more frequent reports of clinical depressive symptoms (x^2^ = 70.63, df = 4, p < 0.001). Similarly, an association was found between the number of life events and clinical depressive symptoms (x^2^ = 40.06, df = 4, p < 0.001). Logistic regression analysis after adjusting for socio-demographic characteristics confirmed that the responders who reported a high number (n = 12–21) of stressful life events during the previous year (OR = 2.64 95% CI: 1.02, 6.83) and a severe degree of stress due to these events (total LESS score > 351, OR = 3.03 95% CI: 1.66, 5.39) were more likely to manifest clinical depressive symptoms.

**Conclusions:**

The high frequency of occurrence of depressive symptoms among Cypriot university students, as well as the strong association with stressful life events, highlights the need for psychological empowerment strategies towards students by institutional counseling services.

## Background

Depression is recognized as a common and debilitating problem amongst student populations, which can affect all areas of functioning including motivation, concentration, feelings of self-worth, and mood [1]. In particular, the prevalence of depressive symptoms among university students is relatively high, ranging from 10.2% to 71.2% [2,3]. In addition, severe depressive symptoms range between 2.3% and 10.9% in this population [4,5]. On one hand, the wide range of estimates in the prevalence of depressive symptoms may to some extent be due to the diversity of the methodological approaches and psychometric tools used, as well as of the socio-cultural characteristics of particular target populations. On the other hand, high rates of depressive symptoms in students might be the result of the multifactorial aetiology of mental disorders, including genetically determined predisposing factors [6,7], socio-demographic factors [8-14], or early life experiences and stressful life events [1,15,16]. Concerning the association between stressful life events and depressive symptoms, the majority of previous findings in the literature suggest that stressful life events have a substantial causal relationship with depressive symptoms [15]. A wealth of published evidence shows that stressful life events are associated with the onset of depressive episodes, and that there is a dose–response relationship. These studies showed that higher levels of stress were related to higher levels of depressive symptoms [15,16,] and demonstrated that the quality of stressful life events was a significant predictor of depressive symptoms [17-24].

According to Wagner et al. [25], stressful life events, either negative (e.g. death of parents, death of a very good friend) or positive (e.g. getting your own car, finding a part-time job), are changes that occur suddenly in one’s life and might have a severe impact on one’s mental health with a risk of depressive symptoms [26]. Stressful life events have been classified according to the degree of their impact on an individual’s life, or of the change in the way one feels about his/her health, or his/her relationship with others [27]. With regards to student populations, the impact of stressful life events and the onset of depressive symptoms may be associated with the students’ personal, psychological, educational, and socio-economic characteristics, affecting their learning ability, concentration, productivity, academic performance, interpersonal relationships [1,27] and quality of life [23]. In fact, the stressors that university students face are different than those faced by their peers who are not in college. In particular, such stressors include the transition to university life, acclimating to a new environment, independently managing the demands of daily life, establishing new social networks, meeting their personal goals [16], academic overload and academic demands, financial pressures, pressure to succeed [1,8,9,12,28] and separation from their usual support network [8,11,12]. When youngsters go to university, they leave behind the people who have been familiar and supportive. Despite the fact that these potentially stressful transitions are universal, they may trigger symptoms of depression to vulnerable individuals [16].

Students who report depressive symptoms may demonstrate a decline in learning abilities and a decrease in the level of information absorbed. Hysenbegasi et al. [29] examined the relationship between depressive symptoms and academic performance in a sample of 330 undergraduate university students. Their results demonstrated that students who reported clinically significant depressive symptoms missed more courses (14.64 versus 2.99 for non- depressed students) and had achieved significantly lower grades than their peers who did not. It was noted, however, that the students who received treatment for depressive symptoms were able to raise their grades to a level similar to their peers [29].

Moreover, financial problems may also be a significant stressor for university students, involved in the occurrence of depressive symptoms and the decline of academic performance [1,14,28]. A study in the United Kingdom investigated the relationship between academic performance, depressive and anxiety symptoms, and particular stressors in a sample of 351 undergraduate students. Over 20% of participants reported major financial problems and that they were unable to cover their daily demands for food supplies. Most importantly, this study demonstrated that financial difficulties had a significant effect on the manifestation of depressive and anxiety symptoms. Additionally, students who were experiencing depressive symptoms and financial problems had lower exam scores compared to students who did not report such issues [1].

Overall, stressful life events faced by university students may have a great impact on their physical and mental well-being, placing them at a high risk of developing both acute and chronic depressive symptoms, the most significant consequence of which is suicide [30]. Therefore, early detection and effective treatment of depressive symptoms may reduce the mental health burden among university populations, improve the longer-term prognosis related to future risk of depression [23], and improve students’ learning ability, productivity, interpersonal relationships, academic performance and quality of life. Thus, targeted interventions towards university students may empower them to develop adaptive coping strategies against stress. In particular, it is suggested that interventions should be focused upon educating students how to decrease the use of emotion focused coping (passive coping strategies) and increase the use of problem solving focused coping (active coping strategies) [31].

### Aim

Although the majority of previous findings in the literature suggest that stressful life events have a substantial causal relationship with the clinically significant depressive symptoms, to date little is known concerning the contribution of the number and severity of recent stressful life events to the prevalence of depressive symptoms. The aim of this study was to investigate (a) the prevalence of depressive symptoms among university students in Cyprus and (b) the association between depressive symptoms and i) the number, and ii) the severity of self-reported stressful life events.

## Methods

### Study population and design

A cross-sectional descriptive correlational study was conducted between November 2010 and May 2011 among all undergraduate students of one of the three public universities in the Republic of Cyprus, the Cyprus University of Technology (CUT). With two thousand forty hundred fifty two (N = 2.452) active students across 10 Departments in 5 Schools, mainly specializing in majors of a technological nature, CUT is the second largest on the island, after the University of Cyprus, offering access to a free education via national examinations. The study was approved by the National Bioethics Committee as well as the Ethics Committee of the University. Prior to data collection, the Heads of all Departments of the University were informed about the purpose of the study and data collection procedures and their consent was acquired. All active undergraduate students from all 10 Departments of the university (1,783 at the time of the study) were eligible to participate, independent of age, gender, and nationality. Students enrolled in postgraduate programmes of study, doctoral programmes or students enrolled in other short-term educational programs were excluded. The final sample consisted of 1,500 undergraduate students (response rate of 85%). The collection of data took place in the students’ classrooms. Among the 283 non-participants, 240 were students who were absent on the day of the survey, 20 students were present but refused to participate and 23 students were excluded from analysis due to missing/incomplete data.

The questionnaire pack included the depression inventory of the Center for Epidemiology Studies (CES-D), the Life Events Scale for Students (LESS) and socio-demographic data, along with an informational sheet explaining the purpose of the study. Questionnaires were distributed to students during class time (either in lecture halls, classrooms, or labs) and verbal consent was obtained. Participation in the study was voluntary and anonymous in order to guarantee confidentiality. After a short briefing, any students who did not wish to participate had the opportunity to leave the classroom. The questionnaires were returned in a collection box in sealed envelopes. The research team coordinated the data collection with the Office for Studies and Student Affairs of the University in order to ensure that it would not coincide with mid-terms, final examinations or any other potentially stressful studies-related activities, such as hospital or industry placements, internships etc.

### Instruments

The instruments (CES-D and LESS) which were included in the questionnaire were written in Greek, which is the native language of the vast majority of the students attending public universities in Cyprus. Although the aforementioned instruments had been validated in previous studies, none of these had been used in the Cypriot population before. Concerning the LESS scale, this was the first time that it was translated into Greek. In addition, while the CES-D scale is largely recognized as a reliable and valid instrument for the screening of depressive symptoms in general and clinical populations in Greece [32], the scale had never been used in Cypriot students before. As a result, some expressions in the items of the scale may have been interpreted differently by different populations, depending on their particular socio-cultural background [33,34]. Thus, translation of the CES-D and the LESS scales into Greek, following relevant guidelines, was performed again for the purposes of the present study [35]. The scales were translated from English to Greek by two independent translators, familiar with the Cypriot culture. The new version of the instruments was compared with the previous one in order to generate a single reconciled version of each instrument, which was then translated back into English. The final version of the instruments was pre-tested with a group of 100 students (pilot study) in order to assess the readability and general comprehensibility. Additionally, as it is described later, the metric properties of each scale were also tested.

#### The depression inventory of the Center for Epidemiology Studies (CES-D)

The CES-D was developed by Radloff [36] to measure the severity of symptoms of depression in community populations. In particular, the scale has been developed to be used as a screening tool of the presence of depressive illness [36]. Items were selected to represent the major components of depression on the basis of the clinical literature and factor analytic studies. Components include depressive mood, feelings of worthlessness and hopelessness, loss of appetite, poor concentration and sleep disturbances, anhedonia, retardation. The scale does not include items which address increased appetite or duration of sleep, psychomotor agitation, guilt and suicidal thoughts [36].

The scale is a composite of 20 items. Four of the items are worded in a positive direction to control for response bias. Subjects are asked to rate each item on a scale from 0 to 3 on the basis of ‘how often you have felt this way during the past week’, 0 = rarely or none of the time (less than 1 day), 1 = some or a little of the time (1–2 days), 2 = occasionally or a moderate amount of time (3–4 days), and 3 = most or all of the time (5–7 days). The CES-D score ranges from 0 to 60. If no more than five items are missing, scores on the completed items are summed up (after reversal of the positive items: 4, 8, 12, and 16); this total is divided by the number of items answered and multiplied by 20. Higher scores indicate more severe depressive symptoms [36].

The reliability and validity of the CES-D have been tested in Africa-American, Asian-American, French, Greek, Hispanic, Japanese, and Yugoslavian populations. Internal consistency as measured by Cronbach’s alpha is high across a variety of populations; generally around 0.85 in community samples and 0.90 in patient samples [36]. Split-half reliability is also high, ranging from 0.76 to 0.85 [36]. Additionally, Radloff [36] has noted that test - retest reliability of the CES-D scale over 2 – 8 weeks has a statistically significant reduction and reported low correlations from 0.32 to 0.67 and between 0.50 and 0,60, which is desirable for a test of symptoms that are expected to show change over time [36]. Studies of African-American versus Anglo-American versus Mexican-American respondents showed no differences in measures of internal consistency reliability [37]. In the current study, the Cronbach’s alpha coefficient for internal consistency was 0.90 and Guttman split-half alpha was 0.89. Cronbach’s alpha values for the subscales were 0.90 (depressive mood), 0.89 (somatic and psychomotor complains), 0.90 (reduced positive affect), and 0.90 (interpersonal difficulties). The average three-week test-retest reliability coefficient for the CES-D total scores was 0.73.

As mentioned earlier, higher scores (in each item and total scores) indicate more severe depressive symptoms. A score of 16 or higher has been used extensively as the cut-off point for clinical depressive symptoms [36]. However, the presence of about 15 - 20% of false positive responds, resulting from the use of this cut-off point, has lead some researchers to suggest a higher cut-off point [38]. In primary care settings a cut-off value of 20–22 is commonly used, as this score increases its specificity of the instrument [39,40]. In the present study, the scores of the severity of depressive symptoms were categorized into 3 groups. In particular, scores of 22 or higher were identified as indicative of clinically significant depressive symptoms. Scores between 16 and 21 were addressed as indicative of the need for a more in-depth assessment or for the treatment of mild to moderate depressive symptoms, while scores of 15 or less were stated as indicative of the absence of depressive symptomatology [36].

#### The life events scale for students (LESS)

The LESS is a checklist of 36 items, specifically designed to assess the severity of distressing situations experienced by university students. Specifically, this scale was designed by Linden [41] to predict the likelihood of disease and illness following exposure to stressful life events. The development of this scale is based on the notion that every life change is followed by feelings of loss and distress, and subsequently by the effort of the individual to adapt to the new circumstances and to regain stability [41]. However, it is worth noticing that the scale’s items do not describe personality traits, nor one’s feelings or perceptions about the events that have occurred. The items included in the scale describe particular events that occurred in one’s life within the past year. The scale assesses the severity of the experienced stress following these events by using Life Change Units (LCU). Each LCU score, assigned to each stressful life event, which might be a major or a minor life situation, positive or negative, reflects the amount of readjustment an individual has to make in order to regain homeostasis.

Linden’s results were based on a study conducted in a sample of Canadian university students, whilst this system of weighting scores was further used in other studies, including that of Clement and Turpin [42] in Britain. While there is some evidence to support the cross-cultural generalizability of the life event weightings [42,43], it is very likely that certain events may be rated differently depending on student’s cultural background [33,34]. Furthermore, the original study was performed 29 years ago (i.e. 1984).

Therefore, it was decided that it was more appropriate to obtain culturally-specific weights from a sample of Cypriot university students. For this purpose, a pilot study was conducted prior to the main study. Since involving undergraduate students in the pilot study would preclude their participation in the main study, it was chosen to include all active postgraduate diploma students. Participation in the study was voluntary and anonymous in order to guarantee confidentiality. The final sample consisted of all postgraduate diploma students (N = 100, response rate 100%, mean age = 24, min = 18, max = 36). Sixty-five percent of the sample included females and 35% males, which is consistent with the male–female ratio of the undergraduate student population. In agreement with the original study, the participants of the pilot study were guided to rate the stressfulness of each of the 36 items of the original checklist on a scale from 0 to 100, in terms of the amount of effort that was required in order to successfully adapt to the particular life event described by the item. The item ‘Death of a parent’ was ranked 100. Weights for each item were calculated by averaging the responses given by the participants. Both the original weights as well as the culturally-specific weights derived in this pilot study were used to calculate the LESS scores of the participants in the main study (Table 1).

**Table 1:** Ranking and weights of LCU for the stressful life events on the LESS scale in the original study and in the Cypriot sample of students

	**Original study by Linden**			**Cypriot students sample**			**Difference**	
**Less scale for university students**	**Rank of the severity**	**LCU M†**	**LCU SD**	**Rank of the severity**	**LCU M‡**	**LCU SD**	**In rank**	**In scores**
Death of a parent	1	100	0.00	1	100	0.00	------	0
Death of the best or of a very close friend	2	87	10.3	2	91	18.2	------	**+**4
Jail term (self)	3	78	20.8	15	62	25.6	−12	−16
Break-up of parent’s marriage/divorce	4	74	22.8	7	72	24.1	−3	−2
Getting kicked out of college	5	72	19.3	5	76	24.8	------	+4
Major car accident (car wrecked, people injured)	6	71	21.7	3	83	23.0	+3	+12
Pregnancy (either yourself or being the father)	7	68	13.7	12	64	29.6	−5	−4
Failing in a number of courses	8	67	24.1	13	64	29.6	−5	−4
Parent losing his/her job	9	66	23.0	11	68	22.9	−2	+2
Major personal injury or illness	10	65	23.8	4	82	20.8	+6	+17
Braking up/loosing contact with a close friend	11	65	23.2	16	62	24.0	−5	−3
Major change of health status in a close family member	12	63	21.1	19	57	24.5	−7	−6
Break-up with boy/girlfriend	13	62	22.9	17	62	25.3	−4	0
Major and/or chronic financial problems	14	60	22.3	6	76	18.4	+8	+16
Moving out of town with parents	15	58	24.7	24	51	27.7	−9	−7
Seriously thinking about dropping college	16	57	27.8	8	72	24.3	+8	+15
Getting an unjustified low mark in a test	17	55	26.3	21	54	27.8	−4	−1
Moving away from home	18	54	24.5	23	52	31.9	−5	−2
Failing in one course	19	53	25.8	9	71	24.0	+10	+18
Switch in a program within the same college or university	20	52	24.2	27	47	29.2	−7	−5
Seeking psychological or psychiatric consultation	21	52	25.7	26	49	25.8	−5	−3
Major argument with parents	22	51	26.5	22	53	22.7	------	+2
Major argument with boy/girlfriend	23	49	23.0	18	60	23.0	+5	+11
Sex difficulties with boy/girlfriend	24	49	25.8	14	64	25.2	+10	+15
Establishing a new steady relationship with a partner	25	44	25.5	28	39	25.4	−3	−5
Minor car accident	26	43	20.4	29	38	25.1	−3	−5
Minor financial problems	27	41	23.2	20	57	24.9	+7	−4
Losing a part-time job	28	40	24.6	10	69	23.4	+18	+29
Getting your own car	29	38	27.2	30	33	28.4	−4	−5
Finding a part-time job	30	37	24.0	31	33	25.3	−1	−4
Change of job	31	35	23.6	25	50	24.4	+6	+15
Minor violation of the law (e.g. speeding ticket)	32	34	23.4	33	27	25.3	−1	−7
Beginning an undergraduate program in the university	33	33	23.4	32	31	26.0	+2	−2
Family getting together	34	30	27.3	34	22	25.0	------	−8
Vacation with parents	35	29	24.4	35	15	18.5	------	−14
Vacation alone/with friends	36	24	23.4	36	14	17.3	------	−10

† In the original study, weights for each event were calculated by averaging (mean and SD) the responses given by the participants. ‡ The weights for each event in this study were calculated in a similar approach to the original study.

### Data analysis

Descriptive statistics for all socio-demographic characteristics, depressive symptoms and life events of the participants were calculated, expressed as appropriately in frequencies, mean values and standard deviation.

Concerning the CES-D scale, each item uses a 0 to 3 response scale; except of the four positively worded items, a higher score in each item indicates greater degree of a depressive symptom. Regarding the items 4, 8, 12, and 16 these are worded positively in part to discourage a response set, thus their scores were reversed by subtracting the score from 3. Items scores were then summed to provide an overall score ranging from 0 to 60. If more than five items on the scale were missing, a score was not calculated. If one to five items were missing, scores on the completed items were summed (after reversal of the positive items); this total was then divided by the number of items answered and multiplied by 20. Levels of clinically significant depressive symptoms were established according to the cut-off points indicated by Radloff [36]. In particular, depressive symptom scores were categorized by cut-off points into 3 groups: (1) Scores less than 15 = Non-depressive symptoms group, (2) Scores between 16–21 = Mild to moderate depressive symptoms group and (3) Scores equal or more than 22 = Clinically significant depressive symptoms group. The association between degree of depressive symptoms and stressful life events was investigated using chi-square tests. Odds ratio (and 95% confidence intervals) of clinically significant depressive symptoms across increasing number of life events and overall LESS score were estimated in logistic regression models before and after controlling for potential socio-demographic confounders. In the absence of theoretical rationale for cut-off points for the LESS scores, we formed categories approximately based on the quartiles of the distribution of scores. Hence, the last category of LESS scores (i.e. 351–1100) for instance comprises of the quartile of participants with the highest scores. The first quartile was further split into two separate categories (0–49 and 50–150) in order to provide separation between those reporting no events (score 0) or only single minor events with those reporting major events, for example, death of parent (score 100), break-up of parents (score 71), major personal injury or illness (score 81) or several more minor events. The Statistical Package for Social Sciences Software (SPSS - version 17) was used to analyze the data. For all statistical tests, p values of 0.05 or lower were considered statistically significant.

## Results

### The socio-demographics characteristics of the sample

The final sample consisted of 1,500 respondents (response rate 85%) from all 10 Departments of the University, of whom 448 were male (29.9%) and 1,052 (70.1%) were female. The mean age of the participants was 20.3 years (SD = 2.1, range: 18–40). Table 2 presents the socio-demographic characteristics of the participants. Most students were living in metropolitan areas (N = 800, 58%), with only 380 (27.6%) of them living in suburban areas and 198 (14.4%) living in rural areas. The vast majority of students were of Cypriot origin (N = 1,415, 94.3%), 67 (4.5%) were Greek, and 18 (1.2%) were foreigners. Only 6.2% (N = 92) of the participants were married or living with a partner, and 0.4% (N = 6) of them were divorced. A significant proportion of students (N = 437, 29%) were employed during the semester. Nearly half of the participants (43.9%) were students in the Faculty of Health Sciences, and specifically in the Department of Nursing, which is by far the largest Department of the University (Table 2).

**Table 2:** The sociodemographic characteristics of the participating students (N = 1500)

	**Frequency (N)**	**Percentage (%)**
**Age**		
*17-20*	933	62.2
*21-24*	502	33.5
*25-40*	65	4.3
**Gender**		
*Male*	448	29.9
*Female*	1052	70.1
**Place of residence**		
*Metropolitan areas*	850	58
*sub-urban area*	452	27.6
*rural area*	198	14.4
**Ethnicity**		
*Cypriot*	1415	94.3
*Greek*	67	4.5
*Other*	18	1.2
**Family status**		
*Single*	1402	93.5
*Divorce*	6	0.4
*Married/living with partner*	92	6.2
**Employment**		
*No*	1063	71
*Yes*	437	29
**Academic year of study**		
*First*	443	29.5
*Second*	426	28.4
*Third*	377	25.2
*Fourth*	254	16.9
**Faculty**		
*Geotechnical sciences and Environmental management*	164	10.9
*Management and Economics*	168	11.2
*Applied Arts and Communication*	219	14.6
*Engineering and Technology*	291	19.4
*Health Sciences*	658	49.3

### Differences in the ranking and weights of the LCU scores of the stressful life events on the LESS scale between the original study and the Cypriot sample of students

Minor differences were observed in the ranking of stressful life events between the sample of the present study and Canadian students from the original survey performed 29 years ago (see Table 1). A difference greater than 10 points in average scores was observed only in 11 out of 36 items. Differences in the magnitude of 15–20 points were observed in 8 out of the 36 LESS items. Regarding these items, the item of jail term moved from position 3 to 15, and for another three items the change was in the range of 10–15. For 25 (70%) of the items the change was only in the range of 0–10 points. As a result of these differences, there were some differences in the ranking, as well. A total of 6 out of the top 10 items were similar between Cypriots and Canadians. Financial problems, losing a job, failing a course or dropping out of university made it to the top 10 among Cypriot students. In terms of the ‘bottom 10’, the picture was very similar between the two samples, with the exception that even minor finance-related problems were rated as more serious among Cypriot students. Generally, it appears that Cypriot students rated finance-related problems as more stressful factors compared to the original study (such as losing a part-time job, which moved from the 28th position to the 10th, major/chronic financial problems, which moved from the 14th to the 6th place, or even changing a job which moved from the 31st to the 25th place). The extent to which this may reflect a true cultural difference or a period effect is unclear. The original study was performed some decades ago while this study was performed just before the financial crisis effects Cyprus. To some extent, this is more likely to reflect the different financial circumstances, since one out of three students (29%) in the present study reported having a day job during semester, indicating that these students’ family could not provide full financial support to them. In contrast, Canadian students seemed to rate as more stressful dealing with problems with the law (e.g. simple breach of the law such as speeding) perhaps because of the fear of punishment. Finally, the Canadian students seemed to report “positive” life events as more stressful, such as the family getting together, vacation with friends, family or alone.

### Prevalence of depressive symptoms and differences between groups among Cyprus university students

The minimum and maximum CES-D values were 0 and 57 respectively (scale range: 0–60). The mean value (± SD) CES-D score was 15.7 (±10.6). The results showed that 25.3% (N = 380) of students suffered from clinically significant depressive symptoms and 18.8% (N = 282) of students suffered from mild to moderate depressive symptoms respectively (Table 3). The prevalence of clinically significant depressive symptoms was statistically significantly higher in females than males (46.6% in females vs. 38.4% in males, x^2^ = 8.53, df = 1, p = 0.003).

**Table 3:** Depressive symptoms scores on the CES-D scale (N = 1500)

	**CES-D scores between 0-15†**	**CES-D scores between 16-21‡**	**CED-D scores** *≤* **22 §**	**Total**
Frequency (N)	838	282	380	1500
Prevalence of depressive Symptoms (%)	55.9	18.8	25.3	100 %
95 % Confidence Interval (%)	53.3-58.4	16.9-20.9	23.1-27.6	

†CES-D scores = 0–15, not indicative of depressive symptoms, ‡ CES-D scores = 16–21, mild to moderate degree of depressive symptoms), §CES-D scored = 22 or higher, severe degree of depressive symptoms.

### Frequency of reported life events

Table 4 presents the frequencies of reported stressful events during the last 12 months in the life of the participants in the main study. The mean number of reported events was M = 5.7 (range 0–21) and standard deviation was SD = 3.2. The most common stressful life events were the last 3 events on the scale. These were minor positive personal life events, and particularly family getting together (N = 639, 42.6%), vacation with parents (N = 498, 33.2%), and vacation alone/with friends (N = 870, 58.0%). One out of three Cypriot students reported finance-related problems (N = 559, 37.3%), losing contact/breaking up with a close friend (N = 499, 33.3%), a major change in the health of a close family member (N = 440, 29.3%) and failing in a number of courses (N = 440, 29.3%). Additionally, 695 students (46.3%) experienced at least one of the top seven stressful life events in the previous 12 months. No student reported experiencing all top seven stressful life events. Finally, 1,091 students (72.7%) experienced at least one moderate to minor life event (i.e. seriously thinking about dropping college, failing in one course, losing a part-time job, or a parent losing the job).

**Table 4:** Frequency of reported stressful life events

	**N**	**%**
1.Death of parent	24	1.6
2.Death of your best or very close friend	166	11.1
3.Major car accident (car wrecked, people injured)	55	3.7
4.Major personal injury or illness	111	7.4
5.Getting kicked out of college	4	0.3
6.Major and/or chronic financial problems	288	19.2
7.Break-up of parent’s marriage/divorce	47	3.1
8.Seriously thinking about dropping college	230	15.3
9.Failing in one course	73	4.9
10.Losing a part-time job	81	5.4
11.Parent losing his/her job	181	12.1
12.Pregnancy (either yourself or being the father)	28	1.9
13.Failing in a number of course	440	29.3
14.Sex difficulties with boy/girlfriend	94	6.3
15.Jail term (self)	58	3.9
16.Losing contact/ breaking up with a close friend	499	33.3
17.Breaing up with boy/girlfriend	355	23.7
18.Major argument with boy/girlfriend	262	17.5
19.Major change of health status in a close family member	440	29.3
20.Minor financial problems	559	37.3
21.Getting an unjustified low mark in a test	370	24.7
22.Major argument with parents	220	14.7
23.Moving away from home	74	4.9
24.Moving out of town with parents	14	0.9
25.Change of job	40	2.7
26.Seeking psychological or psychiatric consultation	46	3.1
27.Switch in a program within the same college or university	261	17.4
28.Establishing a new steady relationship with a partner	238	15.9
29.Minor car accident	307	20.5
30.Getting your own car	341	22.7
31.Finding a part-time job	261	17.4
32.Beginning an undergraduate program in the university	117	7.8
33.Minor violation of the law (e.g. speeding ticket)	308	20.5
34.Family getting together	639	42.6
35.Vacation with parents	498	33.2
36.Vacation alone/with friends	870	58.0

### Associations between degree of depressive symptoms and stressful life events

A positive and statistically significant association was observed between high scores on the LESS scale, indicative of the severity of life stressors in a student’s life, and clinically significant depressive symptoms (x^2^ = 70.63, df = 4, p < 0.001). Similarly, there was an association between the number of stressful life events and clinically significant depressive symptoms (x^2^ = 40.06, df = 4, p < 0.001). Table 5 presents the prevalence of depressive symptoms in terms of the number of reported life events as well as the overall LESS score associated with these events. The prevalence of clinically significant depressive symptoms (as indicated by a score of 22 or above on the CES-D) among the students who did not report any life events in the last 12 months (N = 35, 2.3%) was 20.0%. While the prevalence of clinically significant depressive symptoms did not appear to differ substantially among those who reported experiencing between 1 and 7 events in the last year, there was a clear stepwise increase in the prevalence of clinically significant depressive symptoms among students who reported 8 events or more. Specifically, the prevalence of clinically significant depressive symptoms was twice as high among those who reported as many as 8–11 events (38.5%), while it was as high as 45.1% among those who reported experiencing more than 12 events in the last year. A similar pattern was observed when the analysis was repeated in terms of overall LESS score. The prevalence of clinically significant depressive symptoms was around 17.0% among those with scores in the range of 0–49, but gradually increased at higher LESS scores, reaching 42.8% among the quartile of participants in the highest score category (Table 5). The non-parametric Spearman’s and Kendall’s correlation coefficients between CES-D total score and number of events was: Spearman’s r = 0.116, df = 1498, *p <* 0.001 and Kendall’s tau = 0.115, df = 1498, *p <* 0.001, while for total LESS scores these figures were: Spearman’s r = 0.160, df = 1498, *p <* 0.001 and Kendall’s tau = 0.235, df = 1498, *p <* 0.001.

**Table 5:** Prevalence of clinically significant depressive symptoms (CES-D ≥ 22) by classification of participants in terms of the number of stressful life events and total score on the LESS scale

**Life Events Scale for Students (LESS) (N = 1500)**	**Total**	**Classification of participants according to reported life events on LESS**		**Prevalence of Depressive symptoms (CES-D ≥ 22)**		**X**^**2**^	**DF**	**P value**
		**N**	**%**	**N**	**%**			
**Number of events in LESS**	0	35	2.3	7	20.0	40.62	4	< 0.001
	1-3	339	22.6	62	20.4			
	4-7	753	50.4	176	25.8			
	8-11	288	19.2	99	38.5			
	12- 21	85	4.8	30	45.1			
**Total Score in LESS**	0-49	88	5.9	15	17.0	70.63	4	< 0.001
	50-149	314	21.0	54	17.2			
	150-241	356	23.9	82	22.9			
	242-350	357	23.7	104	29.3			
	351-1100	385	25.5	164	42.8			

The observed associations attenuated but persisted after adjusting for socio-demographic characteristics in multivariable logistic regression models with those reporting the greatest number of stressful life events or the highest total score on the LESS scale more likely to report clinically significant depressive symptoms (Tables 6 and 7, respectively). For instance, students who reported the greatest number of stressful life events appeared 2.64 times (95% CI: 1.02, 6.83) more likely to report clinically significant depressive symptoms compared to those with the lowest scores. Stronger associations were observed with regard to the overall LESS score (which takes into account the severity of the events) than the reported number of events. For instance, the quartile of students with the highest LESS scores were 3.03 times (95% CI: 1.66, 5.39) more likely to report clinically significant depressive symptoms compared to those with the lowest scores. It should be noted that the analyses presented here are based on a cut-off point of 22 or above on the CES-D scale. Repeating the analysis to include those who scored in the mild-to-moderate range on the CES-D (i.e. 16 or more) does not alter our conclusions in terms of the direction or even magnitude of the association with number or severity scores of life events. Specifically, the adjusted Odds Ratios of depressive symptoms (CES-D ≥ 16) across increasing number of events compared to those reporting no events were: 0.60 (95% CI: 0.29,1.23) in those reporting 1–3 events, 0.83 (95% CI: 0.41, 1.66) in those reporting 4–7 events, 1.14 (95% CI: 0.56, 2.33) in those reporting 8–11 events and 2.58 (95% CI: 1.12, 5.93) among those reporting more than 12 events. Similarly, in terms of the LESS scores, a similar stepwise increase in the ORs of depressive symptoms was observed. Other than the slightly tighter confidence intervals (due to higher number of participants scoring above 16), the observed associations are similar. Specifically, compared to those with scores 0–49, the adjusted ORs across categories of increasing scores were 0.73 (95% CI: 0.44, 1.22), 1.26 (95% CI: 0.77, 2.06), 1.55 (95% CI: 0.95, 2.55) and lastly, 3.15 (95% CI: 1.92, 5.17) among the quartile of the participants with the highest scores.

**Table 6:** **Odds ratios (and 95** % **CI) of clinically significant depressive symptoms (CES-D ≥ 22) by the number of stressful life events according to LESS scale after adjusting for all sociodemographic factors as estimated in multivariable logistic regression models**

**Number of events in Less scale**	**Unadjusted**		**Adjusted†**	
	**OR (95%CI) P value**		**OR (95** % **CI) P value**	
**Number of events**				
*0*	1	-----------	1	-----------
*1-3*	1.01 (0.37-2.14)	0.804	0.80 (0.33-1.93)	0.617
*4-7*	1.21 (0.52-2.83)	0.653	1.09 (0.46-2.56)	0.848
*8-11*	2.10 (0.88-4.10)	0.093	1.85 (0.77-4.43)	0.168
*12-21*	3.13 (1.23-7.98)	0.017	2.64 (1.02-6.83)	0.045
**Age**				
*17-20*	1	-----------	1	-----------
*21-40*	1.04 (0.81-1.31)	0.773	1.18 (0.83-1.66)	0.345
**Gender**				
*Male*	1	-----------	1	-----------
*Female*	1.28 (0.99-1.67)	0.050	1.52 (1.12-2.05)	0.007
**Place of residence**				
*Metropolitan areas*	1	-----------	1	-----------
*Suburban or rural areas*	1.18 (0.94-1.50)	0.154	1.22 (0.96-1.56)	0.100
**Ethnicity**				
*Cypriot*	1	-----------	1	----------
*Greek/Other*	1.32 (0.82-2.13)	0.253	1.15 (0.70-1.90)	0.574
**Family status**				
*Single/divorce*	1	-----------	1	----------
*Married/living with partner*	1.28 (0.77-1.90)	0.072	0.96 (0.58-1.60)	0.897
**Employment**				
*Yes*	1	-----------	1	----------
*No*	1.63 (0.90-1.47)	<0.001	1.33 (1.00-1.59)	0.049
**Academic year of study**				
*First*	1	-----------	1	-----------
*Second*	1.37 (1.01-1.86)	0.041	1.30 (0.94-1.78)	0.120
*Third*	1.09 (0.79-1.51)	0.590	0.93 (0.64-1.34)	0.687
*Fourth*	1.14 (0.80-1.63)	0.474	0.94 (0.58-1.55)	0.807
**Faculty**				
*Geotechnical sciences and*	1	-----------	1	-----------
*Environmental management Management and Economics*	1.52 (0.92-2.50)	0.102	1.07 (0.70-1.65)	0.742
*Applied Arts and Communication*	1.46 (0.90-2.34)	0.122	1.53 (1.03-2.29)	0.036
*Engineering and Technology*	1.70 (0.90-2.67)	0.020	1.52 (1.06-2.19)	0.021
*Health Sciences*	1.01 (0.67-1.54)	0.947	1.89 (0.37-2.61)	<0.001

**†**Adjusted for age, gender, ethnicity, place of residence, family status, employment status, year of study, faculty.

**Table 7:** **Odds ratios (and 95** % **CI**) **of clinically significant depressive symptoms (CES-D ≥ 22) by the total score on the life events scale for students (LESS) after adjusting for all sociodemographic factors as estimated in multivariable logistic regression models**

**Total score of events in LESS**	**Unadjusted**		**Adjusted†**	
	**OR (95%CI)**	**P value**	**OR (95** % **CI)**	**P value**
**Score of events**				
*0-49*	1	--------------	1	-----------
*50-149*	1.03 (0.43-1.53)	0.513	0.79 (0.41-1.50)	0.463
*150-241*	1.25 (0.68-2.30)	0.480	1.17 (0.63-2.18)	0.606
*242-350*	1.80 (0.99-3.30)	0.055	1.75 (0.95-3.23)	0.072
*>351*	3.17 (1.75-5.73)	<0.001	3.03 (1.66-5.39)	< 0.001
**Age**				
*17-20*	1	--------------	1	----------
*21-40*	1.04 (0.81-1.31)	0.773	0.78 (0.62-1.24)	0.451
**Gender**				
*Male*	1	--------------	1	----------
*Female*	1.28 (0.99-1.67)	0.050	1.50 (1.10-2.03)	0.009
**Place of residence**				
*Metropolitan areas*	1	--------------	1	----------
*Sub-urban or rural areas*	1.18 (0.94-1.50)	0.154	1.23 (0.96-1.57)	0.094
**Ethnicity**				
*Cypriot*	1	--------------	1	-----------
*Greek/Other*	1.32 (0.82-2.13)	0.253	1.89 (0.54-1.48)	0.662
**Family status**				
*Single/divorce*	1	-----------	1	----------
*Married/living with partner*	1.28 (0.77-1.90)	0.072	1.47 (0.29-1.74)	0.863
**Employment**				
*Yes*	1	-----------	1	----------
*No*	1.63 (0.90-1.47)	<0.001	1.31 (1.01-1.55)	0.050
**Academic year of study**				
*First*	1	--------------	1	-----------
*Second*	1.37 (1.01-1.86)	0.041	1.35 (0.97-1.86)	0.072
*Third*	1.09 (0.79-1.51)	0.590	0.98 (0.67-1.42)	0.915
*Fourth*	1.14 (0.80-1.63)	0.474	1.02 (0.62-1.70)	0.930
**Faculty**				
*Geotechnical sciences and*	1	--------------	1	-----------
*Environmental management Management and Economics*	1.52 (0.92-2.50)	0.102	1.36 (0.81-2.30)	0.241
*Applied Arts and Communication*	1.46 (0.90-2.34)	0.122	1.36 (0.83-2.23)	0.221
*Engineering and Technology*	1.70 (0.90-2.67)	0.020	1.79 (1.12-2.86)	0.016
*Health Sciences*	1.01 (0.67-1.54)	0.947	0.91 (0.60-1.42)	0.702

†Adjusted for age, gender, ethnicity, place of residence, family status, employment status, year of study, faculty.

Finally, in our study, the association between clinically significant depressive symptoms and life events (both number and severity) was statistically significant irrespective of academic year of study (all chi-square values < 0.05, results not shown in detail). As shown in Tables 6 and 7, while in the univariate analyses a higher prevalence of clinically significant depressive symptoms was observed amongst sophomore students compared to freshmen (OR = 1.37, p = 0.041), the association with year of study did not persist after controlling for life events and other socio-demographic variables in multivariable models.

## Discussion

The main finding of the present study was that a high prevalence of depressive symptoms exists among Cypriot university students. The prevalence of clinically significant depressive symptoms (scores 22 or more on CES-D) was 25.3% while as many as 44.1% scored higher than 16 on the CES-D. Several studies to date have shown that university students are at a higher risk for depression, thus our findings are consistent with the majority of previous findings in the literature. International studies from the last five years show that the occurrence of depressive symptoms amongst students range from 10.2% in Switzerland [2] to 71.2% in India [3], with severe depressive symptoms ranging from 2.3% in Brazil [4] to 10.9% in Saudi Arabia [5]. In Europe, research data indicate higher occurrence of depressive symptoms in Eastern countries compared to Western ones (34% in Poland, 39% in Bulgaria, 23% in Germany [12] and 30.4% in United Kingdom [44]). Moreover, in Greece [45], the prevalence has been reported as 52.4%. In the USA and Mexico the prevalence for such symptoms ranges between 16.7% [13] and 36.3% [46], while higher prevalence, in general, is observed in Middle Eastern (21.8% to 39.4% in Turkey [47-49], 39.8% to 53% in Iran, [50-52]) and Asian populations, with the prevalence as high as 46.2% in Malaysia [22], 21.5% to 71.2% in India [3,8], 37.1% in Korea [53], 37.6% in Taiwan [54], and 19.5% to 43.9% in Pakistan [55,56]. The lowest prevalence of depressive symptoms has been reported among the students in Switzerland (10.2%) and in the USA (16.7%) [2,13]. The wide range of the prevalence of depressive symptoms among students in different countries or between diverse samples of students in the same country may, to some extent, be the result of the methodological approach, the psychometric properties of the tools used, and the socio-cultural characteristics of each sample. No closely related research studies on the prevalence of depressive symptoms, or the effect of stressful life events, have been previously conducted among university students in Cyprus; thus there are no data available for comparison. In fact, there is limited data with regards to the prevalence of depressive symptoms among the general population in Cyprus. A recent study among four hundred and sixty-five Cypriot adults of an average age of 53 years has been undertaken by Kiliari et al. [57], in which data collection involved personal interviews via a structured questionnaire. In this study, 3.2% to 5.1% of the participants were found to suffer from depressive symptoms, while the socioeconomic status of the responders was associated with the lifetime prevalence of self-reported depressive symptoms.

Consistent with the majority of the studies in the literature [4,5,12,47,50,56,58-61], the present study reported statistically significant differences in severe clinical depressive symptoms by gender, with higher prevalence among female students. However, a number of studies have found either no differences or the opposite pattern, with male students manifesting higher levels of depressive symptoms than female students [8,11,24,45,62]. Generally, a higher prevalence of depression amongst females has been associated with socio-cultural explanations, including factors related to gender role, as well as with biological and psychological parameters. The impact of personality traits has been examined, with higher levels of distress reported in women [63]. Variations in these findings have also been accounted for by differences in reporting of depressive symptoms, with females being more likely to admit and subsequently to report such symptoms [64].

With regard to stressful life events among university students, the present study revealed significant associations between the number and severity of stressful life events during the latest 12 months and the presence of clinically significant depressive symptoms. Our findings are consistent with the majority of previous findings in the literature that suggest that stressful life events have a substantial causal relationship with the onset of episodes of major depression [15,18,26,65-69]. Additionally, the vast majority of research on the relationship between life stressors and depressive episodes involves occasional, distinct events with particular duration, negative or undesirable content [25]. The relationship between life stressors and risk for depression has been documented for acute and chronic stressors [17,69], as well as for both recent and early life negative events [70]. Moreover, prolonged life stress has been implicated in the first onset of depression [18], recurrence of depression [71] and the exacerbation of depressive symptoms [72].

Moreover, although there are studies that demonstrate an association between severe or major stressful life events and depressive symptoms there exists a number of studies in the last 20 years that have extended their investigation on stress to include the effect of minor stressful events of everyday life. Consequently, there is growing evidence indicating that minor life events often show a stronger relationship to numerous health outcomes, compared to major life events. Research findings concerning depression support that both major and minor stressful life events are associated with the onset of depression, however, minor life events are likely to have an independent and often stronger effect on depressive symptoms than major life events [18,20]. In line with these findings, a large proportion of Cypriot students in the present study experienced minor life events like academic stressors, social stressors, and financial stressors, all of which may place these students at risk of depressive symptomatology. In fact, as many as 72.7% of students experienced at least one minor to moderate life event (i.e. seriously thinking about dropping out of college, failing in one course or dropping out of university, losing a part-time job, or parent losing the job) and one out of three Cypriot students reported finance-related problems, losing contact/ braking up with a close friend, major change of the health status of a close family member and failing in a number of courses. Similar results have been reported in other studies among student population, were certain stressful life events have been associated with higher prevalence of depressive symptoms.

These stressful life events include the relocation and break-up of a significant relationship [23], increased workload, stress and pressure regarding academic demands, pressure to succeed, low academic performance [2,9,12,28,29,55], dissatisfaction with education, perception of low self-efficacy, conflicts between personal and educational demands, low social support, uncertainty about the future [2,11,12,55], low socio-economic level [1,12,14,55], chronic illness or disability [14,23,44,48], history of psychiatric and physical disorder [14,44,47,48,50].

Furthermore, in the present study it was found a strong positive association between the prevalence of clinically significant depressive symptoms and stressful life events, both in terms of the reported number out of 36 events, as well as the total score as measured by the LESS, reflecting the severity associated with these events. The observed association with the number of events attenuated slightly but persisted after adjusting for socio-demographic characteristics in multivariable logistic regression models. The respondents who had reported the greatest number of life events were at least two times more likely to report clinically significant depressive symptoms compared to those who did not report any stressful life events. The association was statistically significant even though the confidence interval was particularly wide due to the small number of students (N = 85) reporting more than 12 events during the last year. Nevertheless, the observed association was not restricted to the group of students with the highest number of events but there appeared to be a stepwise increase in the prevalence of clinically significant depressive symptoms across categories of increasing number of events. There was also a stepwise increase in the prevalence of clinically significant depressive symptoms in terms of increasing LESS scores, with a 3-fold increase in the odds of reporting clinically significant depressive symptoms among the quartile of participants with the highest scores. This is more likely to be due to better exposure classification with the use of the score (rather than the number of events) since it is a function of both the number of events and severity of each event and, as a result it may differentiate better between participants who might report a similar number of events albeit with different severity rating.

To date, little is known concerning the contribution of the number and severity of recent stressful life events to the prevalence of depressive symptoms. However, there is some evidence of a generally linear association between severity and number of negative events and probability of depression [17]. Brown et al. [66] revealed that most severe events (e.g. death of a loved one, divorce, serious illness, losing one’s job) rapidly lead to depressive symptoms and persons who have suffered a severe stressful event were at a four-fold greater risk for developing depression in the subsequent 6 months compared to others who did not suffer a severe stressful life event [66]. Additionally, Kendler et al. [17] supported that the severity of stressful life event can influence depressive symptoms in a number of ways, including triggering a depressive episode, changing the course of depression, leading to increased chance of recurrence and impairing response to treatment; the great majority of major and minor life events associated with the presence of severe clinically significant depressive symptoms occurred within the first month after the event [17]. Additionally, Gillespie et al. [21] showed that the severity and the number of personal stressful life events were associated with depression throughout life [21].

Although, our results are consistent with the majority of previous findings in the literature suggesting an association between stressful life events (major or minor) and depression [15,18,26,65-69], the interpretation of our findings has to be made under the scope of the consideration of the possibility of complex and reciprocal relations between stress and depression [73]. In particular, there exists a substantial amount of support for the stress generation effect in depression, with many studies replicating the original finding [Hammen, 73] that depression is associated with subsequent occurrence of dependent stress [73,74]. Hammen [73] was the first clearly to formulate and test the stress generation model of depression [75]. According to this model, depression-prone individuals are not simply passive respondents to stressful events in their lives, but active agents in the creation of depressogenic life stressors [75]. That is, individuals vulnerable to depression are more likely to experience a higher rate of dependent events, particularly within interpersonal domains. Moreover, these dependent events are to some extent influenced by maladaptive characteristics (e.g., cognitive styles, personality traits) and behaviors of the individual [73-75]. Considering that interpersonal and dependent events, compared to independent ones, seem to be more predictive of depression [18], the generation of dependent life stressors, in turn, may potentially have a role in the maintenance of current depression or increase in the likelihood of subsequent depression onset and recurrence [73].

Finally, the present study adds more evidence to the existing literature [e.g. 1, 9, 15–20, 23, 26] providing new data on the association between stressful life events and depressive symptoms among a special population group, like the students. In the present study we measured the overall stressful experience in terms of 36 life events during the previous 12 months, rather than individual events. We showed that there is an association between clinically significant depressive symptoms and the number of reported events, and in fact, that the association persisted when the severity of these events was included in the measure. In addition, the present study supported further evidence regarding the validity of the LESS scale. Furthermore, the relatively large size of the sample, together with the large number and fields of life events covered in the scale, ensures a reasonably high level of generalizability of the study results. The LESS questionnaire approach to life events measurement possess several advantages compared to interview methods or to studies examining only a small range of specific life events [e.g. studies: 1, 23]. These include ease of administration, suitability for use with large numbers of subjects and covering a large number and different types of events such as major, minor and positive life events.

### Limitations

The above findings need to be viewed in the context of certain methodological limitations. The data collection took place in the students’ classrooms, hence, students that were absent on that day were excluded, along with those who refused to participate, As a result, the observed prevalence of depressive symptoms and the association with stressful life events may be underestimated since it is likely that those who suffer from psychological distress or mental problems are more likely to skip classes. More importantly, the cross-sectional nature of the study does not permit any inference with regard to the direction of the observed association between life events and depressive symptoms. At least with regard to some life events, reverse causality may be at play. For instance, job loss, or failing in one course may be the result rather than the cause of depressive symptoms. Nowadays, research on depression has given increasing consideration to the possibility of complex and reciprocal relations between prolonged stress and depression. Not only does prolonged stress increase risk for depression (i.e. a stress exposure model of depression) but depression, or depressogenic vulnerability, in turn, may also increase susceptibility to stressful events [26,73-75]. Longitudinal studies should aim to explore particular life-related factors that may lead to depressive symptoms. Finally, cross-national comparisons are difficult, thus there is a need for collaborative international studies to investigate the prevalence of depressive symptoms among student populations, across different settings and cultures, employing common psychometric tools and standard methodology. Nevertheless, the large sample and the use of more appropriate and robust instruments (i.e. the CES-D and the student-specific LESS scale) in the present study allow for a more accurate estimation of the occurrence of depressive symptoms and its correlation with stressful life events in the target population. More importantly, in contrast to previous studies, the present study did not focus on specific events but examined the extent to which the number of reported stressful life events, along with the severity of these events was associated with the presence of clinical depressive symptoms. Even though the use of external weights for the estimation of the severity of each event may be viewed as an advantage, nevertheless, the LESS like other ‘checklist methods’ scales has certain disadvantages compared to contextual-based interview methods. The later may allow a more objective assessment of stress severity by the interviewer [76,77]. However, the contextual method of stressful life event assessment is particularly valuable in relation to depression research because bias may arise when depressed individuals report life events, possibly via a pessimistic retrospective style or negative interpretations. Indeed, compared to ‘checklist methods’ whereby the participants ‘checks off’ and subjectively rate the severity of their own life events, contextual methods have been consistently related to fewer response errors due to individual subjectivities, such as mood state [34], personality traits, individual perception, fluctuations in the accuracy of recall and dating of the occurrence of the events [78]. Thus, contextual rating systems have been shown to be more reliable for the exploration of the relationship between stressful life events and the onset of severe depressive symptoms, compared to checklist method [15]. However, the disadvantages of these methods, including the need for trained interviewers and the fact that are time consuming, make them unsuitable for use in large-scale investigations, such as the present study [26]. In addition, Kessler [15] supported that “context” information that is elicited and folded into the threat ratings may itself consist risk factors that account for the association between the reported stressful event and depression [15].

## Conclusion

There is an alarming prevalence of depressive symptoms among Cypriot university students. Additionally, the number and the severity of stressful life events were related to the presence of clinically significant depressive symptoms. There are important implications deriving from the findings of the present study in terms of identifying the most vulnerable students who are in need for psychological empowerment [79]. Most importantly, in view of the relatively high prevalence of depressive symptoms among Cypriots university students, there is a wider need to educate this population how to cope with stressors and depressive symptoms, in order to achieve not only a better quality of life, but an elevated level of performance at individual and institutional level. Additionally, stress reduction among Cyprus students may be important in decreasing the incidence of depressive symptomatology. Interventions (e.g. enhancement of social supportive network, spirituality and effective coping mechanisms) aiming to support students to adjust to their college experience may have a positive result in terms of personal and academic life of vulnerable individuals`. Moreover, education programs in positive health strategies and school counseling programs may be effective ways to reduce and even prevent serious mental health problems (e.g. education workshops or symposia, or even individual consultations). Such programs could assist students to avoid passive coping strategies and provide them the support structures they need to pursue more active strategies. Higher levels of adaptive coping strategies (i.e. cognitive flexibility, strategy-situation fit, and goal attainment) have been found to be associated with higher levels of positive adjustment [17,80] and lower levels of symptoms of depression.

## Competing interests

The authors declare that they have no competing interests.

## Authors’ contributions

The study was jointly designed by all authors and forms part of the work undertaken by SS for his PhD thesis for which AM, NM and MK form the advisory committee. SS organized the collection of data, performed the statistical analysis and prepared the first draft. Each author has made substantial contributions to the conception, design, analysis and interpretation of data and has been involved in drafting the manuscript or revising it critically for important intellectual content and given final approval of the version to be published.

## Authors’ information

^1^SS: PhD (c), MSc, BSc(Hons)Ns, BScEd, Dipl.ED, Dipl.MHN, RGN, RMHN, is a Senior Lecturer in Mental Health Nursing in the Department of Nursing, Faculty of Health Sciences, Cyprus University of Technology. ^2^MA: PhD, MSc, BSc, RGN, is Associate Professor in Nursing Management and Dean in the Faculty of Health Sciences, Cyprus University of Technology. ^3^NM: PhD, MSc, BSc, is Assistant Professor in Health Research Methodology and Biostatistics in the Department of Nursing, Faculty of Health Sciences, Cyprus University of Technology. ^4^KM: PhD, MSc, BSc, RGN, is Assistant Professor (acting) in Mental Health Nursing in the Department of Nursing, Faculty of Health Sciences, Cyprus University of Technology.

## Pre-publication history

The pre-publication history for this paper can be accessed here:

http://www.biomedcentral.com/1471-2458/13/1121/prepub
